# The Hospital World

**Published:** 1911-02-25

**Authors:** 


### The Hospital World.

### Personalia.

We understand that Miss Pratt, St. Augustine, Florida, U.S.A., Councillor Geo. S. Lawson, and Mr. Richard Thornton have been elected Life Governors of the Sunderland Infirmary.

As mentioned in another column, an address has been presented to Her Royal Highness Princess Christian (Princess of Great Britain and Ireland) by St. George's Hospital on accepting the office of President. As this address contains interesting references to the great personalities of the hospital's history, we quote these paragraphs in full : "May it please your Royal Highness,—We, the Governors of St. George's Hospital, beg to tender to your Royal Highness our most grateful thanks for conferring upon us the honour of becoming President of the Hospital in succession to His Present Majesty King George V., who has now become its Patron. St. George's Hospital was founded in 1733, since which time, with the exception of a short period, it has been honoured by the reigning Sovereign being its Patron, and a member of the Royal Family its President. Among its most noted members have been : John Hunter, the founder of modern surgery; Thomas Young, who first established the undulation theory of light; Edward Jenner, the discoverer of vaccination; Sir Benjamin Brodie, who introduced the modern treatment of joint-diseases; James Hope, who established the scientific treatment of heart-disease. Out of five medical men who during the past 100 years have been buried or commemorated in Westminster Abbey, four have been connected with this Hospital. Your Royal Highness's name will ever be associated with your efforts to raise the standard and welfare of the trained nurses of the country, and it is largely due to your Royal Highness's efforts that so great an improvement in this direction has taken place of recent years. That your Royal Highness has seen fit to appoint Her Highness Princess Victoria of Schleswig-Holstein, who has for so long taken an interest in the hospital and its patients, one of your Vice-Presidents, is a source of great gratification to the Governors."

A crowd of supporters attended the recent annual meeting of the Brighton, Hove, and Sussex Throat and Ear Hospital to hear the Bishop of Lewes move the adoption of the report. Its chief feature was perhaps the gaps it chronicled in the ranks of its most prominent supporters. The deaths of Mr. Cresswell Baber, a founder and pioneer of research work (which his linguistic powers allowed him to follow in almost every country of Europe), of Sir Henry Aubrey-Fletcher, of Mrs. Neves, and of Miss Fish remove workers to whom the hospital has owed much in service and in enthusiasm. Let us hope that next year's report will show that the tradition they have set is being carried on with something of their spirit. The principal capital sums received during the year were used to pay off the debt of £615, which, save for the odd sovereigns, is now a thing of the past. It must be added that the late secretary, Mr. Slingsby Roberts, was referred to in a manner worthy of his energy and directness, for his death was a very serious loss to the institution, which lost, in addition to those already mentioned, Mr. George de Paris, the late distinguished chairman of the hospital. This office he had carried on for many years, through good and through evil report, and his example has been followed ably by the group of workers whose record has been noticed above.

